# Poor survival outcomes in HER2-positive breast cancer patients with low-grade, node-negative tumours

**DOI:** 10.1038/sj.bjc.6604940

**Published:** 2009-02-17

**Authors:** S M Tovey, S Brown, J C Doughty, E A Mallon, T G Cooke, J Edwards

**Affiliations:** 1 Glasgow Royal Infirmary, Section of Surgery, Division of Cancer Sciences and Molecular Pathology, Faculty of Medicine, Level 2, Queen Elizabeth Building, 10 Alexandra Parade, Glasgow G31 2ER, UK

**Keywords:** HER2, breast cancer, trastuzumab

## Abstract

We present a retrospective analysis on a cohort of low-grade, node-negative patients showing that human epidermal growth factor receptor 2 (HER2) status significantly affects the survival in this otherwise very good prognostic group. Our results provide support for the use of adjuvant trastuzumab in patients who are typically classified as having very good prognosis, not routinely offered standard chemotherapy, and who as such do not fit current UK prescribing guidelines for trastuzumab.

Human epidermal growth factor receptor 2 (HER2) amplification has become the prototype biomarker for translation of a laboratory discovery through to development of a highly successful individualised biological therapy agent. Slamon *et al* (1987) established HER2 as a poor prognostic marker for survival in breast cancer and developed a monoclonal antibody, trastuzumab, targeted to HER2, as a novel therapy for breast cancer patients. More recently, randomised trials in early breast cancer have shown the clinical benefit of trastuzumab after chemotherapy with significant overall survival benefit over chemotherapy alone (Romond *et al*, 2005; Slamon *et al*, 2006; Smith *et al*, 2007). As a result, trastuzumab has been introduced into routine clinical practice in the UK for HER2-positive patients who have completed their standard adjuvant treatment. Current Scottish and National Institute for Health and Clinical Excellence (NICE) guidelines parallel the herceptin adjuvant (HERA) trial entry criteria, according to which trastuzumab is offered only to those patients who have already received standard chemotherapy regimes as part of their treatment regime.

However, there remains a small subset of HER2-positive patients who are low grade and node negative and who are currently ineligible for trastuzumab treatment as clinically they have been deemed to have no requirement for standard adjuvant chemotherapy. In our region, approximately 25% of HER2 patients are not offered herceptin as they are deemed to be at ‘low risk’ (personal communication). Our study analyses a retrospective cohort of low-grade and node-negative tumours, traditionally classified by Nottingham Prognostic Index (NPI) and Adjuvant! Online as ‘low risk’ to assess whether HER2 positivity affects survival in this otherwise very good prognostic group.

## Methods

### Patients

We have a large cohort (*n*=1351) of breast cancers diagnosed between 1980–2002 with full clinical follow-up (median 6.5 years) and pathological details taken from pathology reports. Tissue specimens from these cancers had been used to create tissue microarray technology (TMA) for research purposes (ethical approval was obtained). The grades of tumours from the cohorts diagnosed in the 1980–90s were reviewed for accuracy by a consultant pathologist (EAM). From this database, we wished to identify a group of patients who would classically be identified as ‘low risk’. We selected all node-negative, grade 1 or 2 cancers (*n*=362) for further analysis.

### Human epidermal growth factor receptor 2 status assessment

Human epidermal growth factor receptor 2 status was identified using currently applicable clinical methodology (Bartlett *et al*, 2003). Dako Herceptest was used to quantify immunohistochemical staining. All 3+ (high-intensity) staining specimens were considered positive. All 2+ (moderate-intensity) staining specimens underwent FISH analysis and those who showed HER2 amplification were also classified as positive by the methods described earlier (Bartlett *et al*, 2003).

### Analysis

SPSS (v15) was used to plot Kaplan–Meier survival curves using breast cancer-specific death as an outcome endpoint (log-rank testing). Cox regression analysis was carried out to evaluate the independence of HER2 in predicting the outcome in conjunction with age, oestrogen receptor (ER), grade, size and endocrine treatment. Cox regression hazard ratios were obtained for studying the effect of HER2 status on breast cancer-specific death in the subcategories split into ER status, age of the patient, and size of tumour.

## Results

### Patient characteristics

There were 362 node-negative, grade 1 or 2 cancers available for analysis. This group contained 90% ER-positive cases, with 71% having size <20 mm. In all, 80% of the patients were aged over 50 years, 10% received chemotherapy and 91% received endocrine therapy (tamoxifen). Table 1 shows the distribution of clinicopathological variables of the good prognostic group split by HER2 status. Fisher's exact test has been used to compare the variables between the HER2 positive and -negative groups. The HER2-positive patients were more likely to be grade 2, ER negative, and were more likely to have received chemotherapy.

### Human epidermal growth factor receptor 2 status and survival

**Figure 1:**
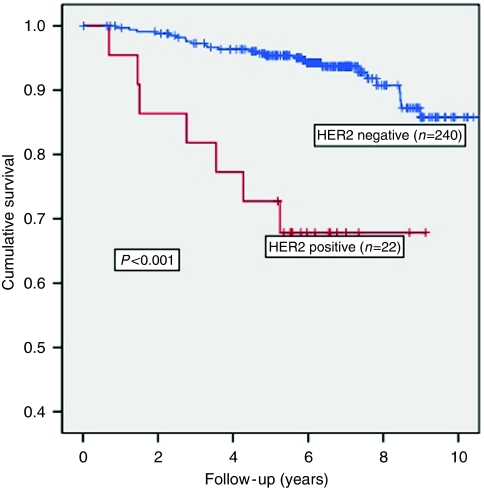
Kaplan–Meier curves for HER2 status. Survival curves showing cumulative survival differences (end point=breast cancer-specific survival) between patients positive or negative for HER2. *P*-value represents log-rank testing of the difference in cumulative survival.

Sixty-one percent of cases were HER2 positive. In univariate log-rank testing, HER2-positive patients were significantly more likely to relapse on tamoxifen, giving 5-year breast cancer-specific survival rates of 68% compared with 96% for the HER2-negative group (*P*<0.001, Figure 1). This significance was retained in Cox regression analysis when analysed alongside grade, size, ER status, age and chemotherapy treatment (*P*<0.001). The overall hazard ratio for HER2 positivity was 5.65 (95% CI 2.4–13.1, *P*<0.001). This reduction in survival in HER2-positive cases persisted when patients were split into subgroups by ER status, tumour size and age (Table 2).

## Discussion

Our results suggest that no HER2-positive patient should be classified as at ‘low risk’. We also suggest that our findings reinforce the importance of having HER2 results available on all patients at multidisciplinary meetings to enable clinicians to make informed decisions on outlook and treatment options.

There have been conflicting reports on the effect of HER2 status in good prognostic groups in the literature. Some have shown similar prognosis in node-negative patients (Paik *et al*, 1990a; Kallioniemi *et al*, 1991; Quenel *et al*, 1995; Press *et al*, 1997; Andrulis *et al*, 1998; Harbeck *et al*, 1999; Schmidt *et al*, 2005) even with small (1–10 mm) tumours (Joensuu *et al*, 2003) or ones with lower grade (Paik *et al*, 1990b). Other papers have not confirmed this (Richner *et al*, 1990; Rosen *et al*, 1995; Ko *et al*, 2007), although care must be undertaken when interpreting earlier studies, which may not have used the currently accepted methods of HER2 testing or are underpowered.

Two large studies, conducted recently, add substantial weight to our findings. In a study of over 2000 node-negative patients (Chia *et al*, 2008), HER2 status was shown to be an independent prognostic factor for disease-free survival in ER-negative patients. Rakkhit *et al* (2009) have shown HER2 to be a significant predictor of disease-free survival in a group of almost 1000 node-negative tumours <1 cm in size.

Our results are in keeping with those from the HERA trial, which suggested that patients with the best prognosis tumours (node negative and size 1–2 cm) had benefit from herceptin, similar to the overall cohort (Untch *et al*, 2008). The 29% of patients in the BCIRG 006 trial who were node negative (Slamon *et al*, 2006) also derived benefits similar to those derived by the whole cohort using trastuzamab, although they were included only if they had another high-risk feature (grade, ER negative over 2 cm).

The persistence of a reduction in survival in our HER2positive or ER-positive subgroup despite endocrine therapy is in keeping with the recent trans-ATAC (Arimidex, Tamoxifen, Alone or in Combination) and BIG1–98 analysis based on HER2 status (Ranganathan *et al*, 2007) (Rasmussen *et al*, 2008) and suggests that we cannot rely solely on adjuvant endocrine therapy (tamoxifen or aromatase inhibitor) in these largely ER-positive patients.

Although this study suggests important findings with respect to HER2 status in good prognosis tumours, we accept the study's limitations with respect to the small number of HER2-positive patients in the cohort. Even within this restricted cohort, HER2-positive patients were less likely to be grade 1 and more likely to be ER negative. In addition, sub-analysis was not performed on tumours <10 mm in size because of the small number of tumours falling into the subgroup.

In conclusion, these results, in the context of other recently reported retrospective studies and adjuvant trials, provide support for the rationale of using adjuvant trastuzumab in this subgroup of patients, who are typically classified as having very good prognosis. These patients may not be routinely offered standard chemotherapy, and as such do not fit the current prescribing guidelines for trastuzumab. A clinical trial to assess the benefit of adjuvant trastuzumab within this group of HER2 patients would resolve this. Whether trastuzumab would be effective alone in these patients (without the potential side effects of standard chemotherapy regimes) deserves investigation. The combination of hormonal therapy and trastuzumab may be particularly beneficial in ER-positive patients, because trastuzumab may overcome the crosstalk between the HER and ER receptors, which is likely responsible for the reduced efficacy of hormonal therapy in this group.

## Figures and Tables

**Table 1:** Clinicopathological details

	**HER2 negative**		**HER2 positive**		**Comparison using**
	**Number**	**Valid %**	**Number**	**Valid %**	Fisher's and χ^2^* (***P*** **values)**
*Grade*					
1	112	32.9	2	9.1	0.018
2	228	67.1	20	90.9	
					
*ER status*					
Positive	270	90.9	13	68.4	0.08
Negative	27	9.1	6	31.6	
Unknown	43		3		
					
*Histological type*					
Ductal	265	78.2	21	95.5	1.139^*^
Lobular	39	11.5	1	4.5	
Other	35	10.3	0	0.0	
Unknown	1		22		
					
*Size*					
T1 (<20 mm)	214	70.9	16	76.2	0.84
T2 (20–50 mm)	88	29.1	5	23.8	
Unknown			1		
					
*Age*					
<50 years	63	18.5	8	36.4	0.252
50–65 years	152	44.7	9	40.9	
65+	125	36.8	5	22.7	
					
*Chemotherapy*					
Yes	27	8.9	6	30.0	0.01
No	276	91.1	14	70.0	
Unknown	37		2		
					
*Endocrine (mainly tamoxifen)*					
Yes	277	91.4	19	100.0	0.382
No	26	8.6	0	0.0	
Unknown	37		3		

Grade=Bloom and Richardson grade; ER status=positive or negative for oestrogen receptor immunohistochemical staining; HER2 status= positive or negative (as defined in text), histological type; ductal=invasive ductal carcinoma; lobular=invasive lobular carcinoma; other includes mucinous, mucoid, etc.; chemotherapy=standard chemotherapy regime at the time of diagnosis. *P* values for comparison of variables between HER2 positive and negative patients using Fisher's and χ^2^^*^ tests.

**Table 2:** Subgroup hazard ratio analysis (Cox regression)

		**Events**				**95% CI**	
	**Number in group**	**HER2 pos**	**HER2 neg (%)**	***P* value**	**Hazard ratio**	**Lower**	**Upper**
Whole cohort	362	7/22 (31.8)	26/340 (7.6)	**0.000**	**5.65**	2.43	13.12
ER positive	283	3/13 (23.1)	17/270 (6.3)	**0.010**	**5.07**	1.47	17.51
ER negative	33	3/6 (50)	6/27 (22.2)	**0.049**	**4.41**	1.01	19.27
Age <50	71	2/8 (25)	3/63 (4.8)	**0.036**	**8.10**	1.14	57.56
Age 50–65	161	3/9 (33.3)	10/152 (6.6)	**0.004**	**6.73**	1.84	24.65
Age >65	130	2/5 (40)	13/125 (10)	**0.033**	**5.09**	1.14	22.66
Size <20 mm	230	5/16 (31.2)	9/214 (4.2)	**0.000**	**8.99**	3.00	26.96
Size >20 mm	93	2/5 (40)	12/88 (13.6)	**0.016**	**6.93**	1.44	33.49
No chemotherapy	290	3/14 (21.4)	13/276 (4.7)	**0.010**	**5.24**	1.49	18.40
Chemotherapy	33	2/6 (33.3)	3/27 (11.1)	0.214	3.11	0.52	18.66
No tamoxifen	26	0	4/26 (15.4)		**n/a**		
Tamoxifen	296	5/19 (26.3)	12/277 (43.3)	**0.000**	**7.29**	2.55	20.78

Hazard ratio=relative increased hazard with 95% confidence intervals from Cox regression analysis for HER2-positive versus HER2-negative cases; percentages=percentage relapse rates in at-risk population.

*P* values are derived from Cox multiple regression analysis with significant hazard ratios shown in bold.
